# Decreased Insulin Clearance in Individuals with Elevated 1-h Post-Load Plasma Glucose Levels

**DOI:** 10.1371/journal.pone.0077440

**Published:** 2013-10-23

**Authors:** Maria Adelaide Marini, Simona Frontoni, Elena Succurro, Franco Arturi, Teresa Vanessa Fiorentino, Angela Sciacqua, Marta Letizia Hribal, Francesco Perticone, Giorgio Sesti

**Affiliations:** 1 Department of Systems Medicine, University of Rome-Tor Vergata, Rome, Italy; 2 Department of Medical and Surgical Sciences, University “Magna Graecia” of Catanzaro, Catanzaro, Italy; GDC, Germany

## Abstract

Reduced insulin clearance has been shown to predict the development of type 2 diabetes. Recently, it has been suggested that plasma glucose concentrations ≥8.6 mmol/l (155 mg/dl) at 1 h during an oral glucose tolerance test (OGTT) can identify individuals at high risk for type 2 diabetes among those who have normal glucose tolerance (NGT 1 h-high). The aim of this study was to examine whether NGT 1 h-high have a decrease in insulin clearance, as compared with NGT individuals with 1-h post-load glucose <8.6 mmol/l (l (155 mg/dl, NGT 1 h-low). To this end, 438 non-diabetic White individuals were subjected to OGTT and euglycemic-hyperinsulinemic clamp to evaluate insulin clearance and insulin sensitivity. As compared with NGT 1 h-low individuals, NGT 1 h-high had significantly higher 1-h and 2-h post-load plasma glucose and 2-h insulin levels as well as higher fasting glucose and insulin levels. NGT 1 h-high exhibited also a significant decrease in both insulin sensitivity (P<0.0001) and insulin clearance (P = 0.006) after adjusting for age, gender, adiposity measures, and insulin sensitivity. The differences in insulin clearance remained significant after adjustment for fasting glucose (*P* = 0.02) in addition to gender, age, and BMI. In univariate analyses adjusted for gender and age, insulin clearance was inversely correlated with body weight, body mass index, waist, fat mass, 1-h and 2-h post-load glucose levels, fasting, 1-h and 2-h post-load insulin levels, and insulin-stimulated glucose disposal. In conclusion, our data show that NGT 1 h-high have a reduction in insulin clearance as compared with NGT 1 h-low individuals; this suggests that impaired insulin clearance may contribute to sustained fasting and post-meal hyperinsulinemia.

## Introduction

Type 2 diabetes mellitus is a metabolic disorder of pandemic proportion, and primary prevention of the disease is an essential strategy to restrain the rapid increase in its prevalence and to control the economic burden on the health care system. There is compelling evidence that lifestyle changes [1], [2] and pharmacological intervention [2]–[4] may prevent or delay progression from impaired glucose tolerance (IGT) to type 2 diabetes. Accurate identification of individuals at increased risk for type 2 diabetes is crucial for intervention programs because it improves the cost-effectiveness of large-scale prevention strategies. It is well established that individuals with IGT are at increased risk for type 2 diabetes compared with individuals with normal glucose tolerance (NGT), but only 35–50% of individuals with IGT develop type 2 diabetes after 5–10 years [5], [6]. Prospective studies have shown that ∼30–40% of individuals who convert to type 2 diabetes have NGT at baseline [5], indicating that the sole use of IGT to identify individuals at increased risk for type 2 diabetes may overlook a large group of individuals that will develop the disease. Recently, it has been shown that a plasma glucose concentration ≥8.6 mmol/l (155 mg/dl) at 1 h during an oral glucose tolerance test (OGTT) can identify individuals at high risk for type 2 diabetes among those who have NGT (NGT 1 h-high) [7], [8]. NGT 1 h-high individuals are characterized by a cluster of metabolic and cardiovascular abnormalities including insulin resistance and reduced β-cell function [9], elevated inflammation markers [10], increased levels of liver enzymes [11], lower plasma insulin-like growth factor 1 concentrations [12], and elevated uric acid levels [13]. Notably, NGT 1 h-high individuals also show signs of subclinical organ damage including common carotid artery thickness [14], vascular stiffness [15], diminished estimated glomerular filtration rate [16], left ventricular hypertrophy [17], and left ventricular diastolic dysfunction [18], all independent predictors of subsequent cardiovascular events.

Insulin clearance is an integrated component of insulin metabolism [19], [20], and may contribute to control cellular response to the hormone by regulating insulin availability, and modulating insulin receptor levels in target tissues of insulin action [21], [22]. The main site of insulin clearance is the liver that removes approximately 80% of endogenous insulin with the remainder being cleared by the kidneys and the skeletal muscle [20]. Receptor-mediated insulin endocytosis is the primary mechanism for insulin clearance from the circulation. Upon insulin binding to its receptor, the insulin-receptor complex is internalized through the formation of clathrin-coated vesicles, and is delivered to the endosomes [20]; the acidification of the endosomes then allows the dissociation of the hormone from its receptor and their sorting in different directions. Most of the internalized insulin is next targeted to lysosomes where it is degraded, whereas a smaller fraction remains intact. Both degradation products and intact insulin are segregated in recycling vesicles and released from cell [23]. Defects in the intracellular processing of insulin have been reported in cells from insulin resistant individuals [24] and reduced insulin clearance has been observed in individuals with IGT [25]. More recently, it has also been demonstrated that reduced insulin clearance predicts the development of type 2 diabetes independently of confounding factors [26]. There is evidence in animal model of fat-induced insulin resistance supporting the idea that decreased insulin clearance may serve as a compensatory mechanism to alleviate β-cell stress from excessive demand [27]. Whether NGT 1 h-high individuals exhibit a decrease in insulin clearance as a compensatory mechanism to preserve β-cell function and maintain glucose levels in the normal range is not known. Exploring this relationship is important to gain insight into potential pathophysiological mechanisms for the development of type 2 diabetes in NGT individuals at increased risk. To examine this issue, insulin clearance was measured in a cohort of non-diabetic White individuals.

## Materials and Methods

### Ethics Statement

The study was approved by the “Comitato Etico Azienda Ospedaliera Universitaria Mater Domini” University of Catanzaro Magna Graecia and by the “Comitato Etico Policlinico Tor Vergata”, University of Rome Tor Vergata and written informed consent was obtained from each subject in accordance with principles of the Declaration of Helsinki.

### Study Subjects

The study group comprised 438 non-diabetic offspring participating to the EUGENE2 project [28], [29] who had one parent with type 2 diabetes. All individuals were White and were consecutively recruited at the Department of Systems Medicine of the University of Rome-Tor Vergata and at the Department of Medical and Surgical Sciences of the University “Magna Graecia” of Catanzaro. All individuals were studied according to a previously described protocol [29]. Exclusion criteria comprised: chronic gastrointestinal diseases or pancreatitis, history of any malignant disease, self-reporting alcohol intake of 2 or more drinks per day, positivity for antibodies to hepatitis C virus (HCV) or hepatitis B surface antigen (HBsAg), and use of medications able to modify glucose metabolism including corticosteroids, glucose-lowering, lipid-lowering and antihypertensive therapy. Smoking was defined as current smoking of at least 1 cigarette/day. On the first day, after 12-h fasting, all subjects underwent anthropometrical evaluation including measurements of body mass index (BMI), waist circumference, and body composition evaluated by bioelectrical impedance. A 75 g OGTT was performed with 0, 30, 60, 90 and 120 min sampling for plasma glucose and insulin. On the second day, insulin sensitivity and insulin clearance were assessed by euglycemic hyperinsulinemic clamp study, as previously described [28], [29]. Briefly, after 12-h fasting, a continuous insulin infusion was initiated at the rate of 40 mU/m^2^ of body surface area per min, after a priming dose, in order to reach and maintain steady state plasma insulin of 618.11±20.84 pmol/l (89±3 µU/ml). Plasma glucose was assessed at 5 minute intervals during the 2-h clamp study by a glucose analyzer. In the study, subjects’ mean plasma glucose concentration during the last hour of the clamp was 5.11±0.22 mmol/l (92±4 mg/dl). Plasma insulin concentrations were measured every 20 minutes during the insulin infusion.

### Calculation

Subjects were classified as NGT [fasting plasma glucose (FPG) <6.11 mmol/l (110 mg/dl) and 2-h post-load <7.77 mmol/l (140 mg/dl)] or IGT [FPG <6.11 mmol/l (110 mg/dl) and 2 h post-load 7.77–11.04 mmol/l (140–199 mg/dl)]. Glucose disposal (M) was calculated as the mean rate of glucose infusion measured during the last 60 min of the clamp examination (steady-state) and is expressed as micrograms per minute per kilogram fat-free mass (M_FFM_) measured with the use of electrical bioimpedance. Insulin clearance was calculated by dividing the rate of insulin infusion by the mean steady-state plasma insulin concentration during the insulin infusion as previously described [30]. The homeostasis model assessment (HOMA) index was calculated as fasting insulin × fasting glucose/22.5 [31].

### Statistical Analyses

Variables with skewed distribution including triglyceride, fasting insulin, 1-h insulin, and 2-h insulin were natural log transformed for statistical analyses; after log transformation the four variables had a normal distribution. Continuous variables are expressed as means ± SD. Categorical variables were compared by χ^2^ test. A general linear model with *post hoc* Bonferroni correction for multiple comparisons was used to compare differences of continuous variables between groups. Relationships between variables were determined by Pearson’s correlation coefficient (r). Partial correlation coefficients adjusted for age and gender were computed between variables.

For all analyses a *P* value ≤0.05 was considered to be statistically significant. All analyses were performed using SPSS software Version 16.0 for Windows.

## Results

Of 438 non-diabetic individuals examined, 64 had IGT and 374 had NGT. A one-hour post-load plasma glucose cutoff point of 8.6 mmol/l (155 mg/dl) during OGTT was used to divide individuals with NGT into two groups: 278 individuals with 1-hour post-load plasma glucose <8.6 mmol/l (<155 mg/dl, NGT 1 h-low), and 96 individuals with 1-hour post-load plasma glucose ≥8.6 mmol/l (≥155 mg/dl, NGT 1 h-high). Table 1 shows the anthropometric and metabolic characteristics of the three study groups. Significant differences between the three groups were observed with respect to gender (higher prevalence of men among NGT 1 h-high as compared with NGT 1 h-low and IGT individuals), and age (NGT 1 h-high and IGT individuals were older than NGT 1 h-low individuals). Anthropometric measures of central (waist circumference) and overall adiposity (body weight, BMI, and fat mass) were higher in IGT individuals as compared with NGT 1 h-low individuals (Table 1). Therefore, all analyses were adjusted for age, gender, and BMI. No significant differences in smoking habit were observed between the three groups of subjects (Table 1).

**Table 1:** Anthropometric and metabolic characteristics of the study subjects stratified according to the glucose tolerance.

Variables	NGT1 h-low (1)	NGT1 h-high (2)	IGT (3)	*P*	*P* 1 vs. 2	*P* 1 vs. 3	*P* 2 vs. 3
*n* (Male/Female)	278 (104/174)	96 (58/38)	64 (33/31)	<0.0001	<0.0001	0.34	0.052
Age (*yr*)	36±10	40±9	43±9	<0.0001*	0.003*	<0.0001*	0.03*
Body weight (*kg*)	80±22	85±18	89±17	0.14**	0.42**	0.05**	0.26**
BMI (*kg/m^2^*)	29.0±7.5	30.3±6.8	32.4±6.1	0.01**	0.16**	0.006**	0.15**
Waist circumference (*cm*)	93±16	97±14	101±11	0.02**	0.22**	0.008**	0.14**
Fat mass (*Kg*)	28±16	28±13	33±15	0.02**	0.78**	0.008**	0.03**
Current smokers (*%*)	27.3	31.3	34.7	0.52	0.48	0.38	0.69
Total cholesterol (*mmol/l*)	4.97±0.95	5.07±0.88	5.51±1.03	0.12	0.73	0.06	0.06
HDL (*mmol/l*)	1.39±0.36	1.32±0.33	1.24±0.33	0.39	0.85	0.17	0.21
Triglycerides (*mmol/l*)	1.20±0.68	1.41±0.85	1.85±1.10	0.003	0.76	0.001	0.005
Fasting glucose (*mmol/l*)	4.77±0.44	5.16±0.49	5.27±0.55	<0.0001	<0.0001	<0.0001	0.24
1-h glucose (*mmol/l*)	6.49±1.22	9.87±1.05	9.82±1.66	<0.0001	<0.0001	<0.0001	0.74
2-h glucose (*mmol/l*)	5.60±1.05	6.21±0.99	8.88±0.88	<0.0001	<0.0001	<0.0001	<0.0001
Fasting insulin (*pmol/l)*	69±48	90±48	118±76	<0.0001	0.04	<0.0001	0.006
1-h insulin (*pmol/l)*	513.9±368	798±548	666±513	<0.0001	<0.0001	0.18	0.009
2-h insulin (*pmol/l)*	375±299	583±472	854±659	<0.0001	<0.0001	<0.0001	0.001
Insulin-stimulated glucose disposal(*µg/min x Kg FFM*)	57.7±27.1	46.6±22.2	41.0±18.3	0.003	0.04	0.001	0.15
Insulin clearance (*ml/min x m^2^*)	567±302	494±201	449±272	0.005	0.01	0.006	0.52
HOMA index	2.3±1.6	2.9±1.8	4.0±2.6	<0.0001	0.03	<0.0001	0.001

Data are means ± SD. Comparisons between the three groups were performed using a general linear model with *post hoc* Bonferroni correction for multiple comparisons. *P* values refer to results after analyses with adjustment for age, gender, and BMI.

* *P* values refer to results after analyses with adjustment for gender.

** *P* values refer to results after analyses with adjustment for age and gender. Categorical variables were compared by χ^2^ test.

A significant decrease in insulin-stimulated glucose disposal, assessed by the hyperinsulinemic euglycemic clamp, was observed in NGT 1 h-high and IGT individuals as compared with NGT 1 h-low individuals, but no differences were observed between the two former groups. Accordingly, HOMA index of insulin resistance was significantly higher in NGT 1 h-high and IGT individuals as compared with NGT 1 h-low individuals, but no differences were observed between the two former groups. The differences in insulin-stimulated glucose disposal remained statistically significant after adjustment for smoking habit (NGT 1 h-high vs. NGT 1 h-low, *P* = 0.05; IGT vs. NGT 1 h-low, *P* = 0.01) in addition to gender, age, and BMI. The metabolic clearance of insulin as obtained during the hyperinsulinemic euglycemic clamp experiment was significantly lower in both NGT 1 h-high and IGT individuals as compared with NGT 1 h-low individuals, but no differences were observed between the two former groups. These differences remained statistically significant when in the general linear model BMI was replaced by waist circumference (NGT 1 h-high vs. NGT 1 h-low individuals, *P* = 0.02; IGT individuals vs. NGT 1 h-low, *P* = 0.005) or by fat mass (NGT 1 h-high vs. NGT 1 h-low individuals, *P* = 0.05; IGT individuals vs. NGT 1 h-low, *P* = 0.01). The differences in metabolic insulin clearance remained statistically significant after adjustment for FPG (NGT 1 h-high individuals vs. NGT 1 h-low *P* = 0.02; IGT individuals vs. NGT 1 h-low, *P* = 0.01); as well as for insulin-stimulated glucose disposal (NGT 1 h-high vs. NGT 1 h-low individuals, *P* = 0.01; IGT individuals vs. NGT 1 h-low, *P* = 0.003) or for HOMA index (NGT 1 h-high vs. NGT 1 h-low individuals, *P* = 0.03; IGT individuals vs. NGT 1 h-low, *P* = 0.05) in addition to gender, age, and BMI.

In univariate analyses adjusted for gender and age, metabolic clearance of insulin was inversely correlated with body weight, BMI, waist circumference, fat mass, 1-hour and 2-hour post-load plasma glucose levels, fasting, 1-hour and 2-hour post-load plasma insulin levels, HOMA index, and positively with insulin-stimulated glucose disposal (Table 2).

**Table 2:** Univariate correlations between insulin clearance and metabolic variables.

	Age and gender adjusted correlations between insulin clearance and metabolic variables	
	Pearson’s correlation coefficient (*r*)	*P*
Body weight (*kg*)	−0.21	<0.0001
BMI *(kg/m^2^)*	−0.19	<0.0001
Waist circumference (c*m*)	−0.17	<0.0001
Fat mass (*Kg*)	−0.27	<0.0001
Total cholesterol (*mmol/l*)	−0.09	0.07
HDL (*mmol/l*)	−0.02	0.74
Triglycerides (*mmol/l*)	−0.08	0.08
Fasting glucose (*mmol/l*)	−0.07	0.11
1-h glucose (*mmol/l*)	−0.18	<0.0001
2-h glucose (*mmol/l*)	−0.17	<0.0001
Fasting insulin (*pmol/l*)	−0.21	<0.0001
1-h insulin (*pmol/l*)	−0.15	0.002
2-h insulin (*pmol/l*)	−0.26	<0.0001
HOMA index	−0.22	<0.0001
Insulin-stimulated glucose disposal (*µg/min x Kg FFM*)	0.10	0.05

## Discussion

In the present cross-sectional study, we provide evidence that individuals with NGT, whose 1-hour post-load plasma glucose is ≥8.6 mmol/l (155 mg/dl), have a reduction in insulin clearance as compared with NGT individuals with 1-hour post-load plasma <8.6 mmol/l. Notably, NGT 1 h-high exhibit a reduction in insulin clearance similar to the one observed in IGT individuals, who are considered at high risk for type 2 diabetes. We found that reduced insulin clearance in NGT 1 h-high and IGT individuals was associated with higher fasting and 2-h post-load plasma insulin levels as compared with NGT 1 h-low individuals.

It has been reported that impaired insulin clearance is a major determinant of hyperinsulinemia in obesity after an oral glucose load [32], and that weight loss, *per se*, primarily increases insulin clearance without affecting insulin resistance or insulin secretion [33]. Therefore, it is possible that the reduction of insulin clearance observed in NGT 1 h-high and IGT individuals may be due to differences in adiposity as compared with NGT 1Vh-low individuals. This possibility is unlikely given that the differences in insulin clearance among the groups remained statistically significant after correction for adiposity measures including BMI, waist circumference or fat mass. These findings are in agreement with results of prior studies carried out on adipocytes isolated from subcutaneous and visceral depots showing that insulin degradation in fat cells was affected by glucose tolerance state rather than by adiposity [34].

It has been suggested that decreased insulin clearance may be a compensatory mechanism to lessen the demand on β-cells in an animal model of insulin resistance induced by high fat diet [27]. Indeed, dogs fed a high fat diet for 12 weeks developed insulin resistance, which was followed by a transient increase in acute insulin response (AIR) to an intravenous glucose tolerance test (IVGTT), and a sustained reduction in insulin clearance to maintain glucose homeostasis [27]. We have previously shown that, as compared with NGT 1 h-low individuals, NGT 1 h-high exhibited a decrease in AIR assessed by IVGTT [9]. Herein, we show that NGT 1 h-high exhibited a reduction in insulin clearance as compared with NGT 1 h-low individuals thus suggesting that decreased insulin clearance may be an adaptive mechanism to preserve β-cells from excessive work to compensate for impaired insulin sensitivity and maintain normal glucose tolerance.

On the other hand, sustained hyperinsulinemia after a meal due to decreased insulin clearance may have other metabolic effects. Recently, we have shown that IGT individuals spend more than 2 hrs per day below the hypoglycemia threshold of <3.89 mmol/l (70 mg/dl) as assessed using a continuous interstitial glucose monitoring [35], and the hypoglycemic episodes were more frequently monitored in the late post-prandial period in a real-life setting. The observation that these asymptomatic hypoglycemic episodes were significantly correlated with insulin levels at 2-h during an OGTT, combined with the present results showing a significant correlation between insulin clearance and insulin levels at 2-h during an OGTT, raises the possibility that hypoglycemia occurring during the late post-prandial period in IGT individuals may be a consequence of a prolonged elevation of insulin due to reduced insulin clearance.

The clinical relevance of characterizing NGT individual at increased risk to develop diabetes is related to the possibility of an early diagnosis and to the potential prevention of the disease and its related complications. Lifestyle changes [1], [2] and pharmacotherapy [2]–[4] in subjects at increased risk for type 2 diabetes, such as IGT individuals, have been demonstrated to be effective in reducing its incidence. It is notable that some pharmacological treatment delaying the onset of type 2 diabetes is also able to improve insulin clearance in IGT individuals [36]. The identification of NGT 1 h-high individuals in a clinical setting could thus have important implications since it could lead to the intensification of lifestyle intervention, and prioritization of pharmacological approaches in these at risk individuals in order to prevent or delay adverse clinical outcomes.

The present study has several strengths including the relatively large sample size, the clinical characterization of subjects performed by a trained staff following a standardized protocol, and the centralized assay of biochemical variables. The present results are also strengthened by the use of gold standard techniques for assessment of insulin sensitivity. Nevertheless, the study has some limitations. First, the cross-sectional design of the study precludes us to draw any conclusion on the role of impairment in insulin clearance in glucose homeostasis deterioration, and, therefore, a cause-effect relationship cannot be firmly established. A second limitation of the present study is that our measurement of insulin clearance is based on a number of assumptions and may be inaccurate to some extent. During the euglycemic clamp a hyperinsulinemic state is induced, and it is possible that the physiological machinery of insulin clearance is saturated; therefore, the metabolic insulin clearance calculated in this study may be lower than what expected at physiological insulin levels. Nevertheless, this method is commonly used for measuring insulin clearance. Another limitation of the present study is that OGTT was only performed once. Although such an approach is common clinical practice, intra-individual variation cannot be taken into account, and some individuals might have been misclassified. Additionally, the present findings might have been influenced by the presence of a family history of diabetes. However, type 2 diabetes has a strong genetic predisposition, and many individuals who develop the disease have a family history of diabetes. Finally, the present results are only based on Caucasian individuals, and generalizing them to other ethnic groups should be done with caution.
